# Lack of modulatory effect of the *SCN5A* R1193Q polymorphism on cardiac fast Na^+^ current at body temperature

**DOI:** 10.1371/journal.pone.0207437

**Published:** 2018-11-12

**Authors:** Masayoshi Abe, Koshi Kinoshita, Kenta Matsuoka, Takahito Nakada, Kimiaki Miura, Yukiko Hata, Naoki Nishida, Toshihide Tabata

**Affiliations:** 1 Laboratory for Biological Information Processing, Graduate School of Science and Engineering, University of Toyama, Toyama City, Toyama, Japan; 2 Department of Legal Medicine, Graduate School of Medical and Pharmaceutical Sciences, University of Toyama, Toyama City, Toyama, Japan; University at Buffalo - The State University of New York, UNITED STATES

## Abstract

*SCN5A* encodes the main subunit of the Na_V_1.5 channel, which mediates the fast Na^+^ current responsible for generating cardiac action potentials. The single nucleotide polymorphism *SCN5A(R1193Q)*, which results in an amino acid replacement in the subunit, is common in East Asia. *SCN5A(R1193Q)* is often identified in patients with type 3 long QT syndrome and Brugada syndrome. However, its linkage to arrhythmic disorders is under debate. Previous electrophysiological studies performed at room temperature inconsistently reported the gain- or loss-of-function effect of *SCN5A(R1193Q)* on the Na_V_1.5 channel. More recently, it was theoretically predicted that *SCN5A(R1193Q)* would exert a loss-of-function effect at body temperature. Here, we experimentally assessed whether *SCN5A(R1193Q)* modulates the Na_V_1.5 channel at various temperatures including normal and febrile body temperatures. We compared voltage-gated Na^+^ currents in *SCN5A(R1193Q)-*transfected and wild-type *SCN5A*-transfected HEK293T cells using a whole-cell voltage-clamp technique. First, we made comparisons at constant temperatures of 25°C, 36.5°C, and 38°C, and found no difference in the conductance density, voltage dependence of gating, or time dependence of gating. This suggested that *SCN5A(R1193Q)* does not modulate the Na_V_1.5 channel regardless of temperature. Second, we made comparisons while varying the temperature from 38°C to 26°C in 3 min, and again observed no difference in the time course of the amplitude or time dependence of gating during the temperature change. This also indicated that *SCN5A(R1193Q)* does not modulate the Na_V_1.5 channel in response to an acute body temperature change. Therefore, *SCN5A(R1193Q)* may not be a monogenic factor that triggers arrhythmic disorders.

## Introduction

*SCN5A* encodes the pore-forming subunit of the human cardiac Na_V_1.5 voltage-gated Na^+^ channel, which is responsible for generating cardiac action potentials [1]. Mutations in *SCN5A* may cause type 3 long QT syndrome (LQT3) and Brugada syndrome (BrS) [1].

*SCN5A(R1193Q)* is a well-known single nucleotide polymorphism (SNP) that causes an R1193Q amino acid replacement in the SCN5A subunit. It is important to evaluate the pathogenicity of this SNP because of its high minor allele frequency (MAF) (0.07 in East Asian populations, Exome Aggregation Consortium). However, previous clinical and epidemiological studies have provided inconsistent results. Some studies identified *SCN5A(R1193Q)* in LQT3 and BrS patients and suggested its possible linkage to sudden cardiac death [2–7]. However, the MAF of *SCN5A(R1193Q)* did not differ between arrhythmic patients, most of whom had structural heart diseases, and healthy controls in Japan (0.063 for both), between the sudden unexpected nocturnal death syndrome cases and the control group in southern China (0.0608 and 0.0476, respectively), and between complete atrioventricular conduction block patients and healthy controls in Korea (0.071 and 0.082, respectively) [8–10].

Electrophysiological characterization in a heterologous expression system could enable the pathogenicity of *SCN5A(R1193Q)* to be understood. Early studies performed at room temperature reported conflicting properties of SCN5A(R1193Q) subunit-containing channels, including persistent conduction during prolonged depolarization possibly leading to gain-of-function, and negative shift of the voltage dependence of inactivation and accelerated decay possibly leading to loss-of-function [11–13]. However, it was unknown whether any of these functional alterations occurred at body temperature. A recent analysis performed at a temperature slightly lower than body temperature (34°C) showed that the density of the voltage-gated Na^+^ current (*I*_Na_) was smaller in *SCN5A(R1193Q)*-transfected cells than in wild-type *SCN5A* [*SCN5A(WT)*]-transfected cells [14]. Furthermore, this study predicted through Q_10_ extrapolation from the data obtained at temperatures of 10–34°C that the reduction of the density of *I*_Na_ mediated by *SCN5A(R1193Q)* channels would be more obvious at normal or febrile body temperature than at lower temperatures. However, this prediction was not tested experimentally.

In the present study, to gain a more direct insight into the pathogenicity of *SCN5A(R1193Q)*, we examined whether it modulated the Na_V_1.5 channel at various temperatures including normal and febrile body temperatures (36.5°C and 38°C, respectively). To this end, we used a whole-cell voltage-clamp technique to compare *I*_Na_ in *SCN5A(R1193Q)*-transfected and *SCN5A(WT)*-transfected HEK293T cells continuously perfused with bath solution at a fixed temperature. Changes in body temperature that occur during cold or hot water bathing or endurance sport may increase the risk of arrhythmic disorders [15, 16]. Thus, we also assessed whether *SCN5A(R1193Q)* modulated the Na_V_1.5 channel in response to body temperature changes by continuously monitoring *I*_Na_ in *SCN5A(R1193Q)*-transfected and *SCN5A(R1193Q)*-transfected HEK293T cells during a gradual change of the bath solution temperature.

## Materials and methods

### Plasmids

This study did not involve human participants, specimens, or tissue samples, or vertebrate animals, embryos, or tissues. Use of the previously cloned human gene (*SCN5A*) was approved by the University of Toyama’s committee (#22–9).

Human *SCN5A* cDNA (GenBank: NM_198056) was subcloned into the *pReceiver-M12* vector containing the N-terminal 3× *FLAG* epitope (GeneCopoeia, Rockville, MD, USA) to create *FLAG-SCN5A(WT)*. *FLAG-SCN5A(R1193Q)* was generated from *FLAG-SCN5A(WT)* using a site-directed mutagenesis kit (Toyobo, Osaka, Japan). cDNA of the Na_V_1.5 channel beta subunit gene (GenBank: NM_001037) was subcloned into the *pReceiver-M12* vector containing the N-terminal 2× *Myc* epitope to create *Myc-SCN1B*.

### Cell culture and transfection

HEK293T cells were cultured in 10% fetal bovine serum-supplemented Dulbecco’s modified Eagle’s medium (Life Technologies) at 37°C in 5% CO_2_. The cells were transiently transfected with a 250 ng mixture of *pCAGGS-EGFP*, *Myc-SCN1B*, and either *FLAG-SCN5A(WT)* or *FLAG-SCN5A(R1193Q)* at a ratio of 1:4.5:4.5 using TransIT-293 (Mirus Bio, Madison, WI, USA). The cells were termed WT and RQ cells, respectively.

### Electrophysiology

We made whole-cell voltage-clamp recordings from the cells as described elsewhere [17]. Briefly, the pipette solution consisted of: 105 mM CsF, 35 mM NaCl, 10 mM HEPES, and 10 mM EGTA. The pH was adjusted to 7.4 at 25°C (7.2 at 36.5°C). The bath solution consisted of: 147 mM NaCl, 4 mM KCl, 2 mM CaCl_2_, 1 mM MgCl_2_, 10 mM HEPES, and 10 mM D-glucose. The pH was adjusted to 7.4 at the temperature of use. Currents were amplified, filtered (5–7.2 kHz), and digitized (100 kHz) using an EPC-10 or 8 amplifier (HEKA, Lambrecht/Pfaltz, Germany). The linear leak was subtracted from the data using a P/10 protocol.

**Fig 1:**
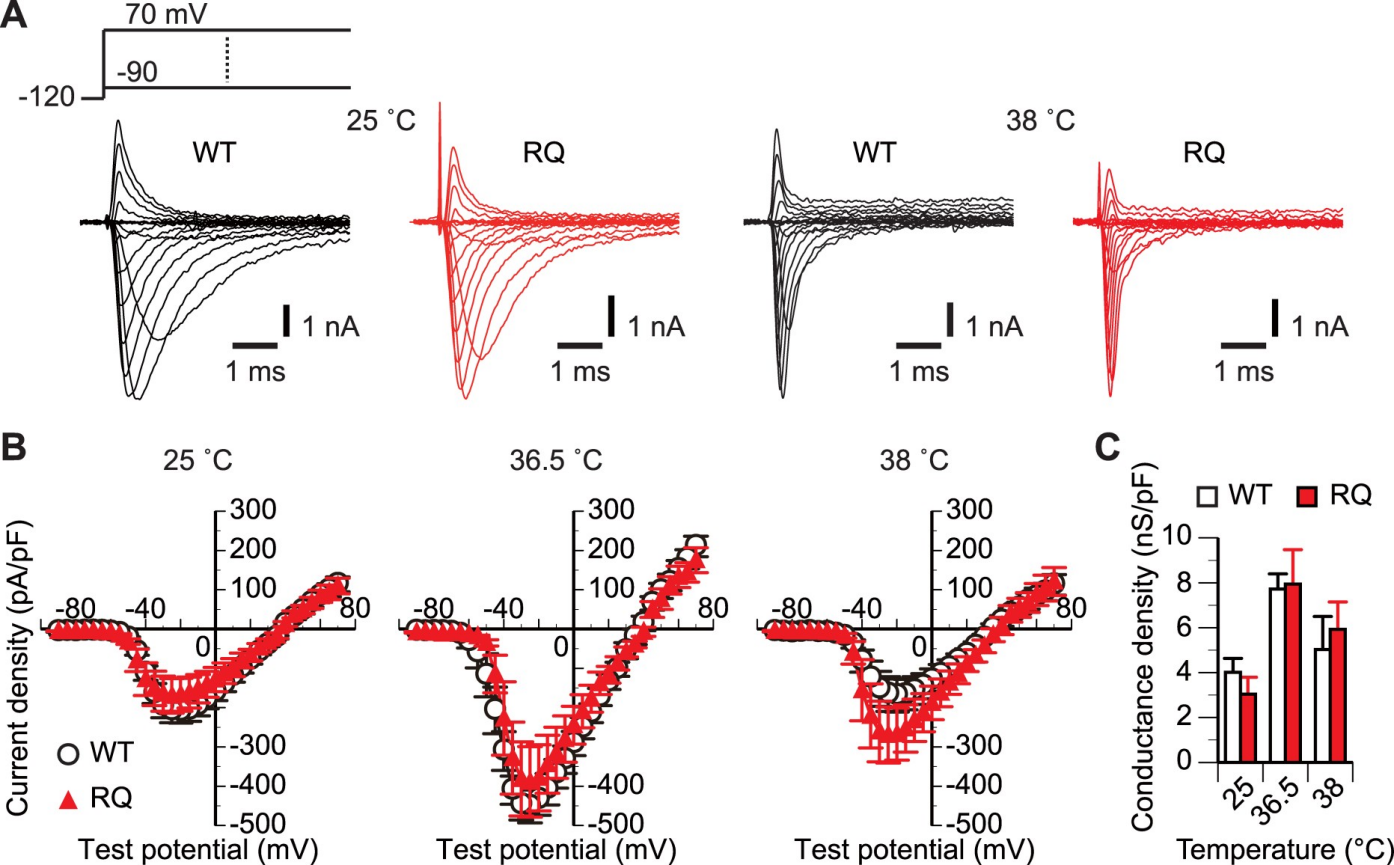
*SCN5A(R1193Q)* did not affect the whole-cell conductance at any tested temperature. (A and B) Representative traces (A) and mean *I-V* plots (B) of *I*_Na_ of WT and RQ cells continuously perfused with bath solutions at the indicated temperatures. Schematic in panel A shows the voltage protocol. The test potential was incremented in 5-mV steps. For simplicity, current responses to some test potentials are omitted from the traces. n, 10–11 WT cells and 9–11 RQ cells for each condition. Error bars indicate ±SEM throughout the figures unless otherwise stated. (C) Comparison of mean whole-cell conductance densities estimated from the slope of the linear region of the *I-V* plot for each cell. p>0.05 between WT and RQ cells for all temperatures (unpaired *t*-test).

**Fig 2:**
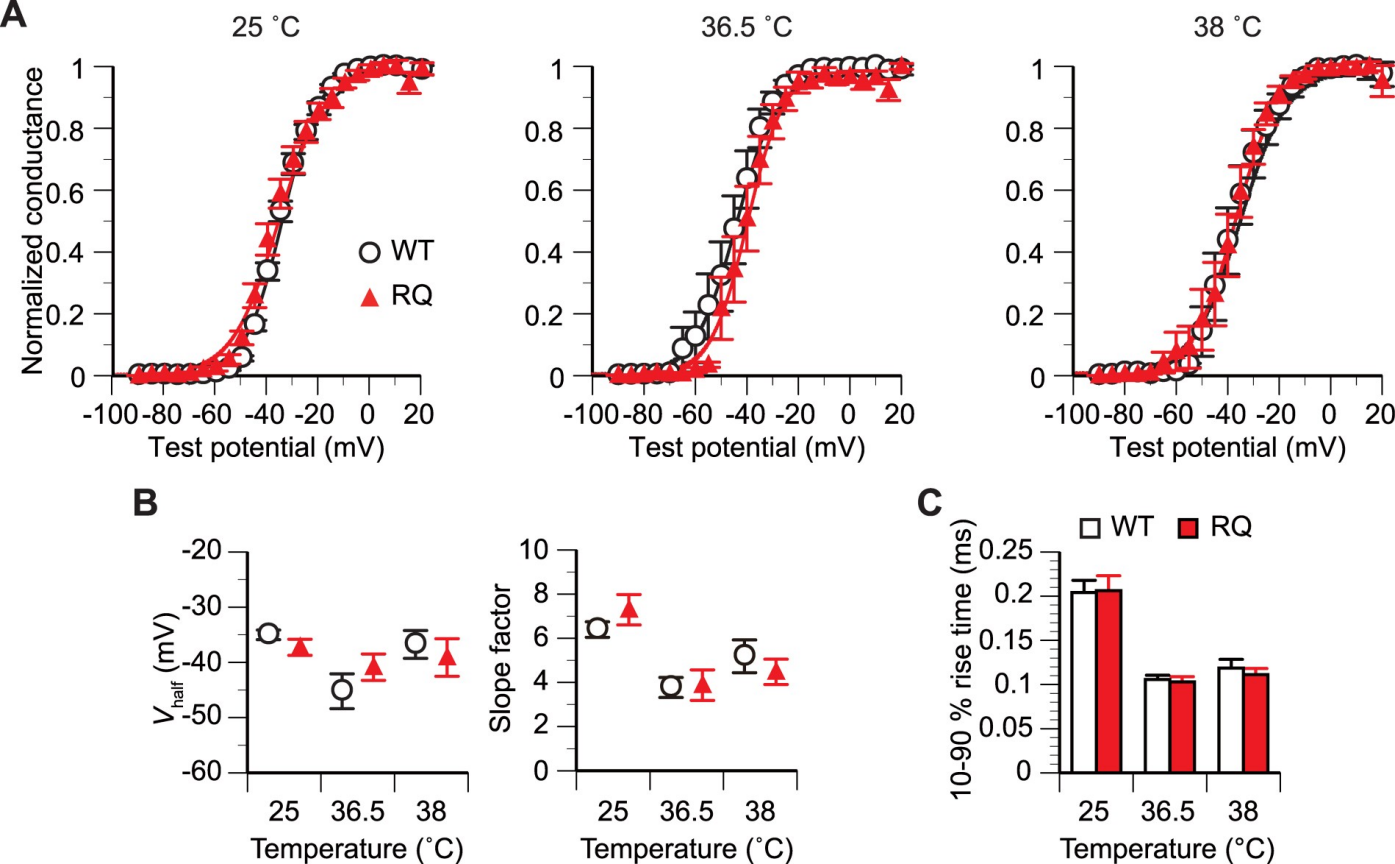
*SCN5A(R1193Q)* did not affect the activation gating at any tested temperature. (A) Mean relative peak *g-V* plots converted from the peak *I-V* plot in Fig 1B. The Boltzmann sigmoidal function was fitted to the data from all examined cells for each condition. n, 10–11 WT cells and 9–11 RQ cells for each condition. (B) Comparison of the mean *V*_half_ and slope factor estimated from the Boltzmann function fitted to the *g-V* plot for each cell. p>0.05 between WT and RQ cells for all temperatures (unpaired *t*-test). n, 10–11 WT cells and 9–11 RQ cells for each condition. (C) Comparison of the mean 10%–90% rise time measured on the current response evoked at a test potential of –20 mV. p>0.05 between WT and RQ cells for all temperatures (unpaired *t*-test). n, 7–10 WT and 7–8 RQ cells for each condition.

**Fig 3:**
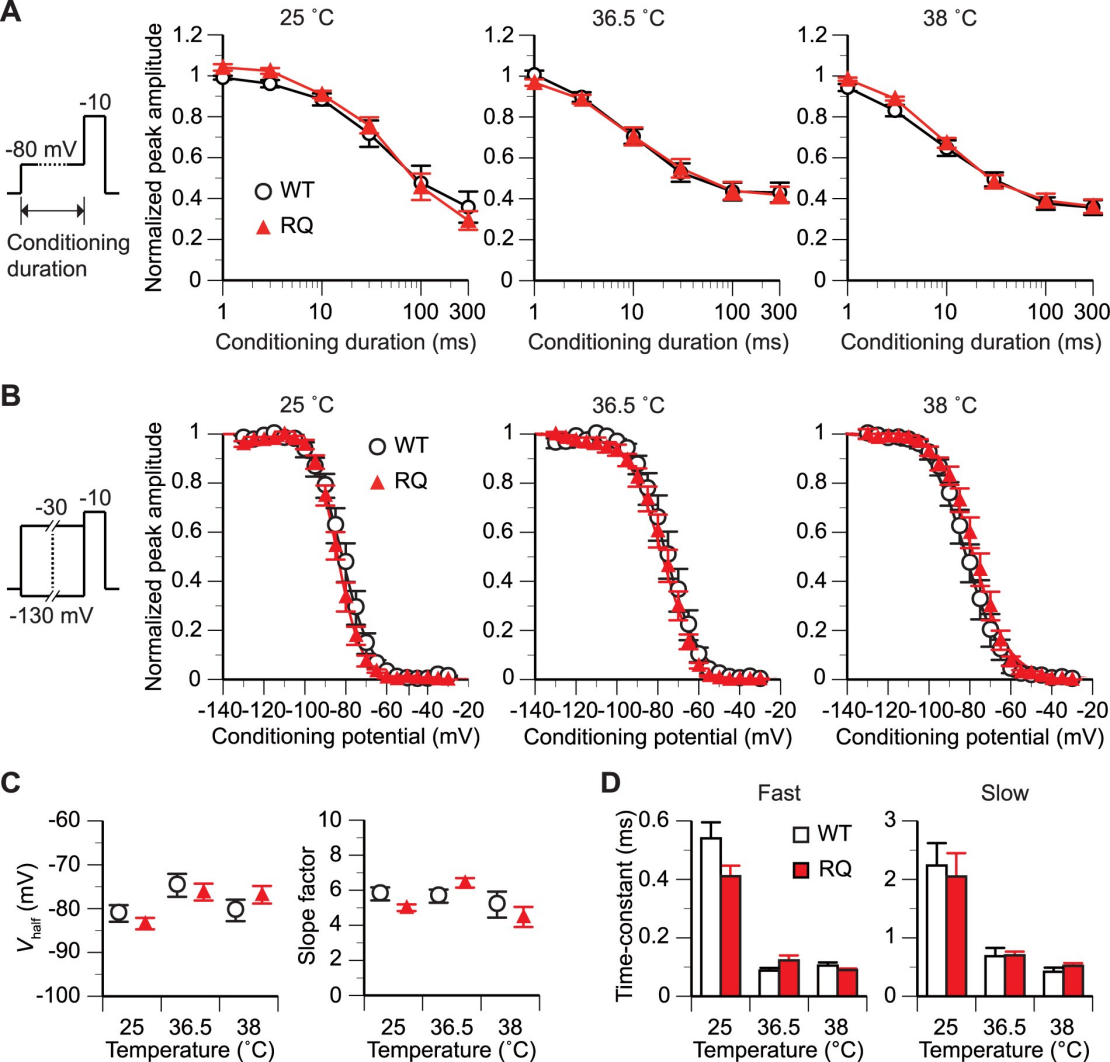
*SCN5A(R1193Q)* did not affect the inactivation gating at any tested temperature. (A) The time dependence of inactivation was measured with a double-step voltage protocol (schematic in panel A; holding potential, -120 mV). The duration of the conditioning step varied from 0 to 300 ms. The duration of the test step was 20 ms. The graph plots the peak amplitude of *I*_Na_ evoked at the test step against the duration. The amplitude is expressed relative to that with a conditioning duration of 0 ms. p>0.05 between WT and RQ cells [repeated measures analysis of variance (rmANOVA)]. n, 5 WT and 5 RQ cells for each data point. (B and C) The voltage dependence of inactivation was measured with a double-step voltage protocol (schematic in panel B; holding potential, -120 mV). The conditioning step was fixed at 500 ms in duration and varied from –130 to –30 mV in voltage. The duration of the test step was 20 ms. (B) The graph plots the relative peak amplitude of *I*_Na_ evoked at the test step against the conditioning voltage. n, 8–10 WT and 8–13 RQ cells for each condition. The Boltzmann sigmoidal function was fitted to the data from all tested cells for each condition. (C) Comparison of the mean *V*_half_ and slope factor estimated from the Boltzmann function fitted to the *g-V* plot for each cell. p>0.05 between WT and RQ cells for all temperatures (unpaired *t*-test). (D) Comparison of mean time constants of the fast and slow components of *I*_Na_ decay. The values were estimated from the double-exponential function fitted to the decaying phase of *I*_Na_ evoked at a test potential of –20 mV for each cell (Fig 1A). p>0.05 between WT and RQ cells for all temperatures (unpaired *t*-test). n, 5–11 WT and 7–8 RQ cells for each condition.

**Fig 4:**
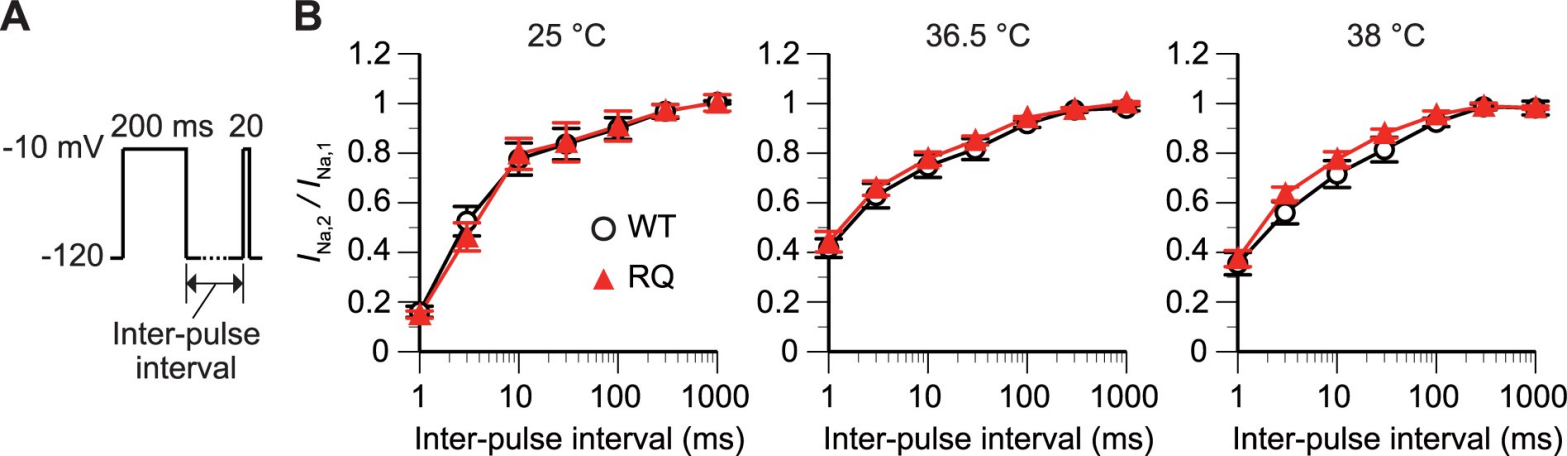
*SCN5A(R1193Q)* did not affect the recovery from inactivation at any tested temperature. (A and B) The time dependence of recovery from inactivation was measured with a triple-step voltage protocol (A). The inter-pulse interval varied from 1 to 1000 ms. The graph (B) plots the ratio of the *I*_Na_ amplitude evoked at the second depolarizing step to the *I*_Na_ amplitude evoked at the first depolarizing step (*I*_Na,2_/*I*_Na,1_) against the inter-pulse interval. p>0.05 between WT and RQ cells (rmANOVA). n, 5 WT and 5 RQ cells for each condition.

In the first set of experiments (Figs 1–4), we perfused the recording chamber with bath solution at a constant temperature throughout the recording. The series resistance (RS) was electronically compensated by 80%. The data were obtained from a different cell group for each temperature.

**Fig 5:**
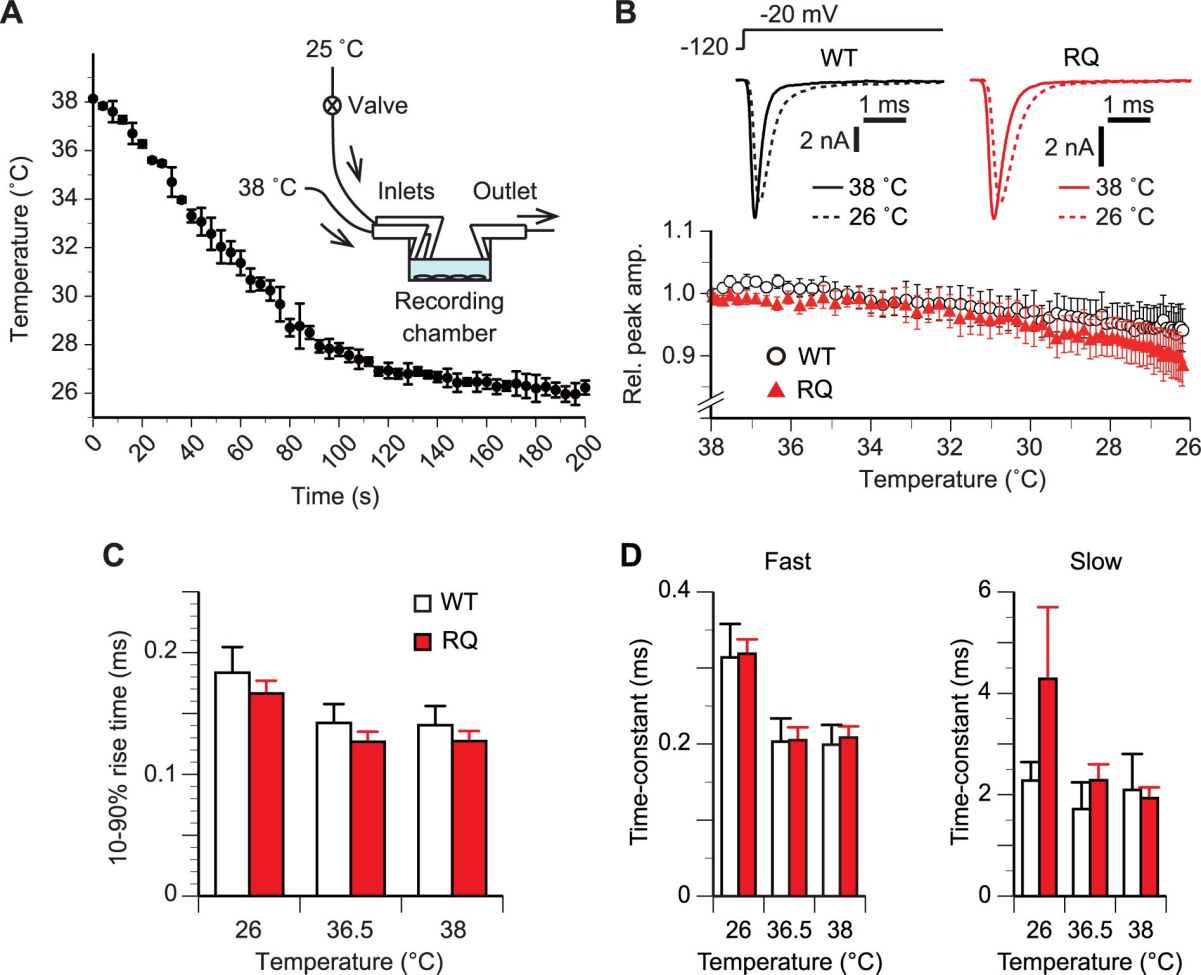
*SCN5A(R1193Q)* did not alter the amplitude or gating in response to an acute change of temperature. (A) Mean time course of the temperature at the center of the recording chamber after starting the additional influx of room-temperature bath solution. Error bars, ±SD. n, 5 trials. Inset, schematic of the experimental set up. The recording chamber was perfused with pre-heated bath solution (38°C) throughout the measurement. (B) Time course of the mean relative peak *I*_Na_ amplitude along with a temperature change from 38°C to 26°C. In panels B–D, *I*_Na_ repeatedly evoked at a test potential of –20 mV in a 2-s interval were analyzed. Holding potential, –120 mV. For each cell, the peak amplitude was normalized to the basal value obtained prior to the lowering of the temperature. p>0.05 between WT and RQ cells (rmANOVA). n, 8 WT and 8 RQ cells. Inset, representative current responses of single WT and RQ cells at labeled temperatures. (C) Comparison in mean 10%–90% rise time. p>0.05 between WT and RQ cells for all tested temperatures (rmANOVA, unpaired *t*-test). n, 8 WT cells and 8 RQ cells. (D) Comparison of mean time constants of fast and slow components of the double-exponential function fitted to the decaying phase of *I*_Na_. p>0.05 between WT and RQ cells for all tested temperatures (rmANOVA, unpaired *t*-test). n, 7 WT cells and 7 RQ cells.

In the second set of experiments (Fig 5), we repeatedly recorded *I*_Na_, varying the bath solution temperature as illustrated in the inset to Fig 5A. First, we made basal recordings, perfusing the recording chamber with a pre-heated bath solution (38°C) at a rate of 1.6 ml/min. Then, we introduced an additional flow (2.2 ml/min) of a room-temperature bath solution (25°C) into the chamber. We measured the time course of the perfusate temperature in mock-up experiments, placing a thermocouple thermometer at the recording site of the chamber. The trial-to-trial deviation in the time course was small (Fig 5A, plot). In actual recordings, the temperature at each time point was estimated from the mean value of the plot. The additional bath solution influx increased the pipette capacitance, causing an error in RS compensation so we did not use the compensation in this experiment. However, this did not greatly affect the measured kinetics of the *I*_Na_. For example, a 10%–90% rise time measured with and without RS compensation was 0.101±0.015 and 0.152±0.030 ms, respectively (n = 8; p>0.05, paired *t*-test; not illustrated).

## Results

### Whole-cell conductance at constant temperatures

In the first set of experiments (Figs 1–4), we compared *I*_Na_ in WT and RQ cells continuously perfused with bath solution at room temperature (25°C), normal body temperature (36.5°C), and febrile body temperature (38°C). We measured the peak *I-V* relations using a single-voltage step protocol with a varied test potential (Fig 1A). The density of whole-cell *I*_Na_ conductance at full activation (Fig 1C) was estimated from the slope of the linear region of the *I-V* plot (Fig 1B). The density was not significantly different (p>0.05, unpaired *t*-test) between WT cells and RQ cells at any tested temperature.

### Activation gating at constant temperatures

The relative *g-V* plots converted from the *I-V* plots show that the voltage dependence of activation was similar between WT and RQ cells at all tested temperatures (Fig 2A). The membrane potential for half-maximal activation (*V*_half_) and the slope factor estimated from the Boltzmann function fitted to the *g-V* plot were not significantly different (p>0.05, unpaired *t*-test) between WT cells and RQ cells at any tested temperature (Fig 2B).

To quantify the time dependence of activation, we measured the time for the current level to increase from 10% to 90% of the peak amplitude (10%–90% rise time) on the trace of *I*_Na_ evoked at a test potential of –20 mV (Fig 2C). There were no significant differences (p>0.05, unpaired *t*-test) in the 10%–90% rise time between WT cells [for 25°C, 36.5°C, and 38°C: 0.204 ± 0.014 ms (n = 10), 0.106 ± 0.005 ms (n = 7), and 0.119 ± 0.010 ms (n = 9), respectively] and RQ cells [0.206 ± 0.017 ms (n = 8), 0.103 ± 0.006 ms (n = 7), and 0.111 ± 0.007 ms (n = 7), respectively].

### Inactivation gating at constant temperatures

In cardiomyocytes under physiological conditions, inactivation occurring at the resting state (~–80 mV) between the action potentials decreases Na_V_1.5 channel availability for subsequent action potentials [18]. Therefore, we next examined whether *SCN5A(R1193Q)* altered such inactivation using a double-voltage step protocol (Fig 3A). As the duration of a –80 mV conditioning step became longer, the relative peak *I*_Na_ amplitude decreased. There was no difference in the dependence of the relative peak *I*_Na_ amplitude on the duration between WT and RQ cells regardless of temperature [p>0.05, repeated measures analysis of variance (rmANOVA)]. This result suggested that *SCN5A(R1193Q)* does not affect the inactivation limiting channel availability.

We also assessed the voltage dependence of inactivation using a double-voltage step protocol with a 500-ms conditioning step that varied in potential (Fig 3B). The plots of peak *I*_Na_ amplitude against conditioning potential were similar between WT and RQ cells at all tested temperatures. Moreover, the *V*_half_ and slope factor of the plot did not differ significantly (p>0.05, unpaired *t*-test) between WT cells and RQ cells at any tested temperature (Fig 3C).

To quantify the time dependence of inactivation, we measured the time constants of the decaying phase of *I*_Na_ evoked at a test potential of –20 mV (Fig 1A). The decaying phase was well fitted by a double exponential function. There were no significant differences (p>0.05, unpaired t-test) in the time constant estimated from the fast component of the fitted function between WT cells [for 25°C, 36.5°C, and 38°C: 0.540 ± 0.055 ms (n = 11), 0.080 ± 0.009 ms (n = 5), and 0.102 ± 0.012 ms (n = 6), respectively] and RQ cells [0.411 ± 0.036 ms (n = 8), 0.128 ± 0.019 ms (n = 7), and 0.093 ± 0.005 ms (n = 7), respectively] (Fig 3D). Additionally, there were no significant differences (p>0.05, unpaired *t*-test) in the time constant estimated from the slow component between WT cells [for 25°C, 36.5°C, and 38°C: 2.239 ± 0.382 ms (n = 11), 0.686 ± 0.142 ms (n = 5), and 0.419 ± 0.071 ms (n = 6), respectively] and RQ cells [2.049 ± 0.400 ms (n = 8), 0.700 ± 0.064 ms (n = 7), and 0.522 ± 0.046 ms (n = 6), respectively] (Fig 3D).

### Recovery from inactivation at constant temperatures

We assessed the time dependence of recovery from inactivation using a triple-voltage step protocol (Fig 4A). *I*_Na,2_/*I*_Na,1_ (see legend of Fig 4 for definition), an index of the degree of recovery, increased with the inter-pulse interval (–120 mV) (Fig 4B). There was no difference in the dependence of *I*_Na,2_/*I*_Na,1_ on the interval between WT and RQ cells regardless of temperature (p>0.05, rmANOVA). This result suggested that *SCN5A(R1193Q)* does not affect recovery from inactivation.

### Amplitude during a temperature change

In the second set of experiments (Fig 5), we evaluated how the channels alter their functional properties in response to an acute change of temperature (Fig 5A). To detect gating alterations occurring along with a temperature change, we repeated single-voltage step stimulations (–20 mV) in a short interval (2 s).

As the temperature decreased, the *I*_Na_ amplitude gradually decreased (Fig 5B). The temperature dependence of the amplitude was not significantly different between WT and RQ cells (p>0.05, rmANOVA).

### Activation gating during a temperature change

We measured the 10%–90% rise time on the data used in the analysis in Fig 5B. As the temperature decreased, the rise of *I*_Na_ slowed down (Fig 5B and 5C; for simplicity, only the data at temperatures near 26°C, 36.5°C, and 38°C are illustrated). There was no significant difference in the temperature dependence of the 10%–90% rise time between WT and RQ cells (n = 8 for each cell group; p>0.05, rmANOVA) (Fig 5C). There was also no difference (p>0.05, unpaired *t*-test) in 10%–90% rise time at temperatures near 26°C, 36.5°C, and 38°C between WT cells (0.184 ± 0.021 ms, 0.142 ± 0.016 ms, and 0.140 ± 0.016 ms, respectively; n = 8) and RQ cells (0.166 ± 0.010 ms, 0.127 ± 0.008 ms, and 0.127 ± 0.008 ms, respectively; n = 8) (Fig 5C).

### Inactivation gating during a temperature change

We next measured the time constants of the decaying phase on the data used in the analysis in Fig 5B. As the temperature decreased, the decay of *I*_Na_ slowed down (Fig 5B–5D). There was no significant difference in the temperature dependence of the time constant of the fast or slow component between WT and RQ cells (n = 8 for each cell group; p>0.05, rmANOVA) (Fig 5D). There was also no difference (p>0.05, unpaired *t*-test) in the time constant of the fast component at temperatures near 26°C, 36.5°C, and 38°C between WT (0.314 ± 0.044 ms, 0.203 ± 0.031 ms, and 0.199 ± 0.026 ms, respectively; n = 7) and RQ cells (0.319 ± 0.019 ms, 0.205 ± 0.017 ms, and 0.208 ± 0.015 ms, respectively; n = 7) (Fig 5D). Similarly, there was no difference (p>0.05, unpaired *t*-test) in the time constant of the slow component at temperatures near 26°C, 36.5°C, and 38°C between WT (2.28 ± 0.36 ms, 1.72 ± 0.53 ms, and 2.09 ± 0.71 ms, respectively; n = 7) and RQ cells (4.29 ± 1.41 ms, 2.28 ± 0.32 ms, and 1.93 ± 0.21 ms, respectively; n = 7) (Fig 5D).

## Discussion

### *SCN5A(R1193Q)* does not modulate the Na_V_1.5 channel at normal or febrile body temperatures

A similarity in whole-cell conductance density between WT and RQ cells at 36.5°C and 38°C (Fig 1C) suggested that *SCN5A(R1193Q)* does not reduce the cell surface expression or unitary conductance of the Na_V_1.5 channel. This directly indicates that *SCN5A(R1193Q)* does not cause the previously predicted loss of channel function that could manifest at normal or febrile body temperatures [14]. Moreover, similarities in the voltage and/or time dependences of activation, inactivation, and recovery from inactivation between WT and RQ cells at 36.5°C and 38°C (Figs 2–4) demonstrated that at normal and febrile body temperatures, *SCN5A(R1193Q)* does not cause the gating abnormalities that were previously reported to occur at room temperature [11–13].

In this study, cells were cultured at a normal body temperature and then exposed to a test temperature during the recording session. Thus, the total time of conditioning to the febrile body temperature prior to measurement was up to ~1 h. We therefore cannot exclude the possibility that long-term (over several hours) exposure to a febrile body temperature could produce a difference(s) in gating and/or membrane trafficking between WT channels and those containing SCN5A(R1193Q) subunit.

### *SCN5A(R1193Q)* does not modulate the Na_V_1.5 channel in a temperature-dependent fashion

Some *SCN5A* mutations were shown to cause channel malfunctions that manifest only near body temperature. For example, *SCN5A(T1620M)* right-shifts the voltage dependence of activation and accelerates inactivation at 32°C [19], while *SCN5A(F1344S)* also right-shifts the voltage dependence of activation at 40.5°C [20]. Moreover, the V1340I mutation in the *SCN5A* splice variant lacking Q1077 showed a reduced current density at 32°C and 37°C [21]. Such temperature-dependent channel malfunctions may underlie the development of arrhythmic disorders in patients with fever [20, 21].

By contrast, *SCN5A(R1193Q)* did not alter the voltage or time dependence of activation, inactivation, or recovery from inactivation at any tested temperature (Figs 2–4). These gating processes were slower at 25°C than 36.5°C and 38°C (Figs 2–4). This temperature dependence was commonly observed in WT and RQ cells, and suggests that *SCN5A(R1193Q)* does not modulate the Na_V_1.5 channel in a temperature-dependent fashion.

The slow-down of activation and inactivation was also observed during a gradual lowering of temperature (Fig 5), although the extent of slow-down was similar between WT and RQ cells. This suggests that *SCN5A(R1193Q)* does not modulate the Na_V_1.5 channel in response to an acute change of temperature. It is noteworthy that differences between the rates of activation and inactivation at body temperature and room temperature measured during a gradual temperature change (Fig 5C and 5D) were smaller than those measured at constant temperatures (Figs 2C and 3D). A possible explanation is that it takes several minutes for cellular mechanisms influencing the rate of the gating processes to adapt to a new temperature.

Taken together, our findings suggest that *SCN5A(R1193Q)* does not modulate the Na_V_1.5 channel at body temperature. These results together with the previously reported similarity in the MAFs of *SCN5A(R1193Q)* between the arrhythmic patients and the healthy controls [8–10] indicate that *SCN5A(R1193Q)* may not be the monogenetic cause of arrhythmic disorders.
